# The Relationship between E-Health Literacy and Health-Promoting Behaviors in Nursing Students: A Multiple Mediation Model

**DOI:** 10.3390/ijerph18115804

**Published:** 2021-05-28

**Authors:** Sunghee Kim, Jihyun Oh

**Affiliations:** 1 Red Cross College of Nursing, Chung-Ang University, Seoul 06974, Korea; sung1024@cau.ac.kr; 2 Department of Nursing, Daejeon University, Daejeon 34520, Korea

**Keywords:** health-promoting behaviors, e-health literacy, self-care agency, online health information-seeking behaviors, nursing students

## Abstract

The availability of a wide range of online health-related information on the internet has made it an increasingly popular source of health information, particularly for people in their 20s. This study aimed to explore possible multistep and indirect pathways of association between e-health literacy and health-promoting behaviors through social media use for health information, online health information-seeking behaviors, and self-care agency among nursing students. The study included 558 nursing students from three different universities in South Korea. Data were collected using structured questionnaires from 2 August to 29 August, 2019. The results show that e-health literacy had a significant direct effect on health-promoting behaviors through the three mediators. Moreover, the overall model explained 46% of the total variance in health-promoting behaviors. Based on these findings, it is necessary to introduce interventions that improve e-health literacy and develop a strategy to promote healthy behaviors. It is also necessary to develop programs to improve e-health literacy competency in nursing students. Moreover, health interventions that improve health-promoting behaviors should be developed, and research to evaluate the effect of the interventions should be conducted.

## 1. Introduction

With the rapid development of information and communication in the Fourth Industrial Revolution (i.e., the digital revolution), people have become increasingly interested in health management through the acquisition and use of health information from various digital sources.

According to a 2018 survey on the status of internet use by the Korea Internet and Security Agency, 91.5% of individuals over three years of age use the internet in Korea. It was found that internet usage was 99.9% for teens, people in their 20s, and people in their 30s. The main purpose of using the internet is obtaining data and information, which accounts for 89.1% of all use [1]. As such, it is thought that college students acquire a range of information, from simple to more specialized knowledge, through the internet. The continuous increase in the number of people who actively search for and use health information through the internet has created the concept of “e-health” [2]. In 2016, the World Health Organization (WHO) mentioned electronic health (e-health) concerning people checking health information through technologies such as the internet in an article [3]. Additionally, Norman and Skinner stated that e-health literacy is the ability to search for, understand, and evaluate health-related information on the internet, as well as to transfer and apply knowledge to deal with and solve health problems [4]. Online health information is searched for mostly by people with a keen interest in health and a positive attitude toward seeking information about health and preventive behavior. This makes them more inclined to actively participate in health-promoting behaviors leading to positive health outcomes [5]. 

Health-promoting behavior includes far more than just disease prevention, such as behaviors that complement healthy lifestyles. These behaviors include activities such as assuming responsibility for personal health, participating in physical activities, and acquiring good nutritional habits [6].

**Figure 1:**
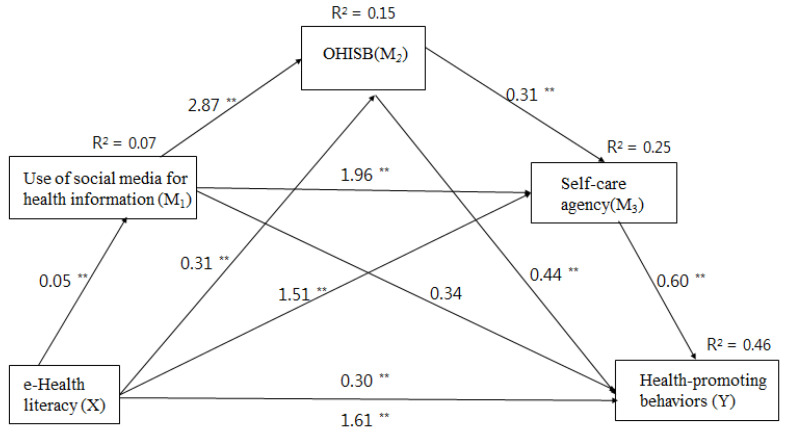
The results of the multiple mediation model testing social media use for health information, online health information-seeking behaviors, and self-care agency as mediators of the effect of e-health literacy on health-promoting behaviors. ** *p* < 0.01.

In order to improve health-promoting behaviors, it is necessary to first identify its influencing factors. In previous studies, these included self-efficacy, self-esteem, perceived health status, optimism, and pessimism. In recent years, however, the concept of health literacy as an influencing factor of health-promoting behavior in individuals has attracted significant attention [7,8]. Pender [9], for instance, explained self-care as an influencing factor, while Orem [10] described an individual’s ability to perform self-care as self-care agency. So [11] categorized self-care agency into six factors: cognitive skills, physical skill, decision-making and judgment processes, information-seeking behavior, awareness of self-control, attention, and value of self-management. Studies have noted that groups with a high level of e-health literacy search for health information on the internet and have a more positive attitude toward such information [12,13]. E-health literacy levels showed the relationship between information-seeking behavior and cognitive aspects, which was one of the self-nursing competencies proposed by So [11]. Furthermore, prior studies indicate that e-health literacy may affect social media use, online health information-seeking behaviors, self-care agency, and health-promoting behaviors [13,14,15,16,17]. However, they have not yet examined the “pathways” of association between e-health literacy, social media use for health information, online health information-seeking behaviors, self-care agency, and health-promoting behaviors in nursing students. The current study proposes a multiple mediation model, which states that e-health literacy affects health-promoting behaviors directly and indirectly via three mediators (Figure 1). 

As health communication managers, nurses play an important role in educating patients and their families on how to access e-health information and evaluate its reliability [14]. Additionally, nursing students themselves must also possess a high level of e-health literacy for their own health and nursing work, where they are in charge of health communication. College years are the most crucial in regard to health because desirable lifestyles and healthy behaviors can be easily adopted. Thus, putting health-promoting behavior into practice is crucial at this age to ensure positive lifelong health [15]. Furthermore, nursing students, who become healthcare professionals when they graduate, not only have the responsibility to positively influence others’ health but also have the opportunity to become role models for health promotion [18]. Therefore, it is of paramount importance that health-promoting behaviors are instilled in nurses. For this, it is necessary to explore the effect of e-health literacy level on health-promoting behaviors among nursing students, especially because existing research on this topic is insufficient.

Based on the existing literature, first, we hypothesized that higher e-health literacy positively affects health-promoting behaviors in nursing students. Second, we hypothesized that e-health literacy resulting from social media use for health information would further improve online health information-seeking behaviors and self-care agency, which in turn positively affects health-promoting behaviors.

Therefore, using a multiple mediation model, the current study aimed to evaluate how e-health literacy (X) directly and indirectly affects health-promoting behaviors (Y) via social media use for health information (M_1_), online health information-seeking behaviors (M_2_), and self-care agency (M_3_).

## 2. Materials and Methods

### 2.1. Study Design and Participants

This study employed a descriptive survey that tested the mediating role of social media use for health information, online health information-seeking behaviors, and self-care agency in the relationships between e-health literacy and health-promoting behaviors. To obtain relevant information, questionnaires were developed and reviewed by the authors based on previous studies. The questionnaires were then distributed to 570 nursing students from the first year to the fourth year from three different universities. Of a total of 570 participants, 558 completed questionnaires were considered for the final analysis after 12 participants who submitted incomplete responses were excluded, representing a response rate of 97.8%.

### 2.2. Variables

#### 2.2.1. Self-Care Agency

Self-as-Carer Inventory (SCI) was developed by Geden and Tylor [19] and was revised and translated into Korean by So [11] to measure self-care agency. The instrument comprises 34 items that are scored on a six-point Likert scale, ranging from 1 (very accurate) to 6 (very inaccurate). The possible scores range between 34 and 204, where the higher the score indicates better the evaluated self-care agency (i.e., the higher the self-care competence). The Cronbach’s α of this instrument for the current study is 0.95, which represents high reliability.

#### 2.2.2. Social Media Use for Health Information

To measure social media use for health information, participants were asked a single question: “In the past 1 month, which of the following types of sites have you used to look up or share health information?” The response options were as follows: (1) web-based blog (2) social networking site (e.g., Facebook, Twitter), (3) online chat room or community, and (4) online group forum, which were responded to by 1 = yes, 2 = no.

#### 2.2.3. Online Health Information-Seeking Behaviors

Inspired by previous studies, online health information-seeking behaviors were measured using an instrument developed by Park and Lee [20,21,22]. The questionnaire comprised 13-items using a five-point Likert scale ranging from 1 (strongly disagree) to 5 (strongly agree). A higher score indicates better online health information-seeking behaviors. Cronbach’s α for the current study is 0.89, showing high reliability.

#### 2.2.4. E-Health Literacy

E-health literacy refers to an individual’s competence in searching for desired health information on the internet, comprehending and evaluating such information, and utilizing the health information in solving his/her health problems. The e-Health Literacy Scale (eHEALS), originally developed by Norman and Skinner [4] (and revised by Lee et al. [23]), was used to assess this competency. In this scale, 8 items were rated on a five-point Likert scale, ranging from 1 (strongly disagree) to 5 (strongly agree). The higher the mean scores, the higher the level of e-health literacy. Cronbach’s α for the current study is 0.93, showing high reliability.

#### 2.2.5. Health-Promoting Behaviors

Health-promoting behaviors were measured using the Health Promoting Lifestyle Profile (HPLP-Ⅱ), developed by Walker et al. [24] and translated into Korean by Seo [25]. Comprising 52 items, this questionnaire included 6 subdomains (health responsibility, physical activity, nutrition, spiritual growth, interpersonal relations, and stress management). Each of the items was evaluated on a four-point Likert scale (1: never; 2: sometimes; 3: often; 4: routinely). The higher the mean scores, the higher the level of health-promoting behavior performance. Cronbach’s α for this scale was 0.92 in Seo’s study [25] and is 0.94 in this study, showing high reliability.

### 2.3. Data Collection

Data were collected from 2 August to 29 August, 2019. The survey was directly distributed to 570 nursing students from three universities after obtaining their written consent. To collect data from nursing students, the authors sent the questionnaires to the dean of the nursing colleges at three universities. The authors visited the three universities and described the purpose of the study to the nursing students after receiving consent from the deans. All participants provided written informed consent before participation and could withdraw from the research at any time. The survey took around 15–20 min to complete. Surveys returned by 12 of the 570 participants were discarded due to being incomplete; thus, 558 responses (a 97.8% response rate) were considered for this study.

### 2.4. Study Analysis

The data were analyzed using SPSS version 23.0 statistical software (IBM Corp., Armonk, NY, USA) to calculate descriptive statistics along with Pearson’s correlation matrix. A serial multiple mediation analysis (Model 6) of the SPSS PROCESS macro [26] was used to test the hypothesis that social media use for health information, online health information-seeking behaviors, and self-care agency acts as a serial mediator of the relationship between e-health literacy and health-promoting behaviors. A bootstrapping method was used to estimate the 95% bias-corrected confidence interval (BC CI) for the indirect effects of each mediator [27]. When the 95% confidence interval does not include zero, it is considered to have significant indirect effects. 

### 2.5. Ethical Considerations

This study was approved by the Institutional Review Board of the Daejeon University (1040647-201904-HR-013-02). Participants who consented to participate in the study were informed of the study’s purposes and procedure. They signed a written informed consent form before participating in the survey. Furthermore, they were informed about the benefits and risks of participation and were reminded that they could withdraw from the study at any time. Moreover, they were informed that the data would only be used for research purposes and that anonymity and autonomy would be guaranteed. 

## 3. Results

### 3.1. Participants’ General Characteristics

Of the 570 participants, 558 were included in the analysis after completing the questionnaire (response rate = 97.8%). The participants’ general characteristics have been provided in Table 1. Most participants were female (88.4%) and aged 17–43 years with an average age of 20.3 years (SD = 2.2). Academically, 29.4% were freshmen, 23.1% were sophomores, 23.3% were juniors, and 24.2% were seniors. Most (60.8%) were unaffiliated with religious groups. Further, 31.0% of participants used the internet for less than two hours per day on average. Over half of them (74.7%) reported that they spent more than four days per week searching for health-related information online. Lastly, 55.4% of the participants perceived themselves as having a good health status. 

Table 1 provides the mean scores for self-care agency, e-health literacy, social media use for health information, online health information-seeking behaviors, and health-promoting behaviors. The mean score for self-care agency is 156.17 (SD = 20.17), the mean score for e-health literacy is 30.53 (SD = 5.20), for health information-seeking behaviors, it is 36.61 (SD = 9.93), and for social media use for health information, it is 2.22 (SD = 1.10). The overall mean score for health-promoting behavior is 2.50 (SD = 0.44). In the health-promoting behavior subscale, the mean scores for interpersonal support and spiritual growth are the highest, whereas the one for physical activity is the lowest. 

### 3.2. Correlations among Self-Care Agency, E-Health Literacy, Social Media Use for Health Information, Online Health Information-Seeking Behaviors, and Health-Promoting Behaviors

Self-care agency showed statistically significant positive correlations with e-health literacy (r = 0.45, *p* < 0.001), social media use for health information (r = 0.26, *p* < 0.001), online health information-seeking behaviors (r = 0.29, *p* < 0.001), and health-promoting behaviors (r = 0.64, *p* < 0.001). E-health literacy showed statistically significant positive correlations with social media use for health information (r = 0.26, *p* < 0.001), online health information-seeking behaviors (r = 0.25, *p* < 0.001), and health-promoting behaviors (r = 0.37, *p* < 0.001). Social media use for health information showed statistically significant positive correlations with online health information-seeking behaviors (r = 0.36, *p* < 0.001), and health-promoting behaviors (r = 0.25, *p* < 0.001). The correlation between online health information-seeking behaviors and health-promoting behavior was also positive at a statistically significant level (r = 0.39, *p* < 0.001; Table 2).

### 3.3. Mediating Effects

In the present study, bootstrapping methods were used in the serial multiple mediation analysis to test whether the indirect effects of e-health literacy on health-promoting behavior through social media use for health information, online health information-seeking behaviors, and self-care agency were significant. The overall effect of e-health literacy on health-promoting behaviors was found to be significant (B = 1.61, SE = 0.16, t = 9.61, *p* < 0.001) (Table 3). The serial mediation model’s results showed that e-health literacy had a significant direct effect on health-promoting behaviors (B = 0.30, SE = 0.15, t = 1.97, *p* = 0.048). The overall indirect effect of e-health literacy on health-promoting behaviors through the three mediators (i.e., social media use for health information, online health information-seeking behaviors, and self-care agency) was found to be significant (B = 1.31, SE = 0.13, BC CI (0.04, 1.58)). Moreover, as displayed in Table 3, the bootstrapped indirect effect of e-health literacy on health-promoting behaviors through social media use for health information and self-care agency was significant (B = 0.06, SE = 0.02, BC CI (0.01, 0.12)); similarly, social media use for health information and online health information-seeking behaviors was significant (B = 0.07, SE = 0.02, BC CI (0.03, 0.11)), and online health information-seeking behaviors and self-care agency was also significant (*B* = 0.06, SE = 0.02, BC CI (0.02, 0.11)). Additionally, the bootstrapped indirect effect of e-health literacy on health-promoting behaviors via online health information-seeking behaviors alone was significant (B = 0.14, SE = 0.04, BC CI (0.06, 0.24)), as was self-care agency (B = 0.91, SE = 0.11, BC CI (0.70, 1.14)). However, the paths from social media use for health information to health-promoting behaviors were not found to be significant (CIs included zero). Figure 1 shows the standardized path coefficients of the proposed serial multiple mediation model, indicating the direct path coefficient between all constructs.

Based on the results, the overall model explained 46% of the total variance in health-promoting behaviors. In addition, this study found that the greater the e-health literacy, the more it would encourage social media use for health information; further, the better the online health information-seeking behaviors and the higher the self-care agency, the better the health-promoting behaviors.

## 4. Discussion

The purpose of this study was to investigate whether e-health literacy affects health-promoting behaviors and whether social media use for health information, online health information-seeking behaviors, self-care agency mediate this relationship. It attempted to provide basic data for developing educational strategies and nursing intervention programs to encourage health-promoting behavior among nursing students.

In this study, the self-care agency of nursing students was 156.17 ± 20.17 points on average (based on 204 points), which was higher than the 137.00 ± 21.95 points found in So’s study when developing a self-care agency measurement tool [11]. Additionally, e-health literacy, social media use for health information, and online health information-seeking behaviors all scored higher than average. This is because, with the development and generalization of smart information devices, anyone can easily access and use health-related information on the internet. The reason nursing students have a high level of e-health literacy is perhaps that they are already acquainted with significant amounts of health information from studying nursing, and apparently, have improved health behavior indicated by their e-health literacy level [28]. The score of the health-promoting behavior for this study’s participants was 2.50 on a four-point scale, indicating a moderate level. By the scores from the health-promoting behavior subscale, interpersonal support (3.15), spiritual growth (2.87), and stress management (2.55) ranked highly. However, physical activity scored the lowest (2.10), indicating that nursing students had low exercise or physical activity. According to recent statistics from the National Statistical Office, the percentage of respondents who reported regular exercise in the 20–29-year age group was as low as 36.8%, which supports our findings [29]. Low health responsibility requires that young people must pay more attention to their own health, constantly learn about health, and seek advice or help from health experts when necessary [30]. A total of 55.4% (n = 309) of the participants of this study perceived themselves as being healthy or very healthy. However, levels of physical activity and health responsibility of health-promoting behavior were low, indicating that there was a difference between health care and awareness and health behavior practice. In a previously conducted study, the level of health-promoting behavior among female college students was higher in the areas of interpersonal relations and self-actualization than in physical activity, which was similar to the results of this study [31]. These results indicate that nursing students are more interested in interpersonal relationships and mental health than learning about physical activity or health or improving overall health and seeking advice from experts. Therefore, there is a need for measures that promote practical health management that maintain their interest in mental health, improve their physical activity levels, and encourage them to pay attention to their overall health status, and seek professional support if needed.

### 4.1. Correlations Between Self-care Agency, E-health Literacy, Social Media Use for Health Information, Health Information-seeking Behaviors, and Health-Promoting Behaviors

Self-care agency showed a significant positive correlation with e-health literacy, social media use for health information, health information-seeking behaviors, and health-promoting behaviors. E-health literacy had a significant positive correlation with social media use for health information, health information-seeking behaviors, and health-promoting behaviors. A study that investigated the mediators of health literacy in HIV patients [32], which included knowledge and behavioral skills related to HIV, found that self-management was promoted in HIV patients via health literacy, suggesting that there is a relationship between e-health literacy and self-care agency. A study by Lee and Oh [33] also reported a correlation among health literacy, self-care agency, and health-promoting behaviors, and explained how health literacy enhances self-care agency. This is interpreted as a result of promoting health-promoting behavior. Therefore, it is necessary for a nursing college student, who will become a nurse in the future, to understand the importance of self-care so that they can take responsibility for and manage the health issues of not only themselves but also others. Furthermore, it is believed to be necessary to develop a systematic education program that can maintain and improve self-care agency. In other studies, groups with high e-health literacy levels were more likely to seek health information online than groups with low levels of e-health literacy, while having a positive attitude toward health information available on the internet, thereby supporting the results of this study [34]. Additionally, the findings of this study, are consistent with the results of another study [18] that reported that the higher the health information-seeking behavior of nursing students, the better their health-promoting behaviors. Social media has an essential place in the lives of both patients and nurses, and has positive as well as negative impacts [35,36]. It enhances health-related knowledge, improves health outcomes, and aids in the decision-making process regarding healthcare [37,38]. Healthcare workers, especially nurses, play a crucial role in fulfilling the patients’ needs [39]. Therefore, to promote health-promoting behaviors, nursing students need a better understanding of the advantages and disadvantages of using social media so that they can identify incorrect or potentially harmful health-related misinformation on social media, which can exacerbate the health of the patients and themselves. Moreover, nursing educators need to consider educational strategies for promoting accurate and acceptable health-related information and identifying reliable information from the internet. If necessary, nursing educators can design, develop, and implement educational plans for social media users.

### 4.2. Mediating Effects

In this study, the overall effect of e-health literacy on health-promoting behaviors was direct and significant. Through self-care agency, social media use for health information, and health information-seeking behaviors, the indirect effects of e-health literacy on health-promoting behaviors were significant. Health information-seeking and self-care agency through social media use for health information were found to have significant effects on health-promoting behavior. Health information-seeking influences health-promoting behaviors by the mediating of self-care agency. E-health literacy improves health information-seeking behavior, which can ultimately lead to health-promoting behavior and health outcomes [40]. Finally, this study found that the higher the e-health literacy, the more it will promote social media use for health information, health information-seeking behaviors, and self-care agency. This will lead to better health-promoting behavior by raising motivation and intention for it. Moreover, a study involving Taiwanese university students also founded [17,41] that e-health literacy was a significant predictor of health-promoting behaviors, such as diet, exercise, and sleep. Therefore, the use of health information obtained from the internet affects the self-care agency and health behavior of individuals [42].

In a study involving college students, it was suggested that groups with high levels of e-health literacy are more likely to search for health information on the internet and actively engage in good health behavior [26]. This indicated that although health information can be acquired through various media, medical personnel were specifically relied upon to provide specialized health information in the past, and this affects individuals’ health-promoting behavior. Thus, the results of this study, which revealed the factors that have a significant influence on health-promoting behavior, are similar to a study by Hong [43], which reported a positive relationship between searching for health information through the media and preventive behavior for cancer screening in adults. This finding was in line with Moon et al. [44], who believed that health information-seeking behavior through active media, such as the internet, improves health. Choi [45], similarly, reported that health-promoting behavior is affected by e-health literacy and health information-seeking behavior. Further, a previous study that investigated e-health literacy in Chinese older adults found that high e-health literacy was associated with positive health behavior [46].

Therefore, for nursing students, e-health literacy influences health-promoting behaviors, and self-care agency, social media use for health information, and health information-seeking behaviors mediate this relationship. Patients, as well as nursing students, use social media for web-based health information. Online health information can improve patients’ overall health outcomes and increase their knowledge [37,47,48]. However, online health information is sometimes scientifically incomplete or inadequate. Physicians are worried about patients feeling threatened and being misled by wrong or inaccurate information [49]. Health-related information on social media and the internet represents a challenge mainly because it can be inaccurate. Therefore, nursing students should effectively and critically evaluate the quality of online health information when caring for patients and themselves. As such, nursing students should be provided with opportunities to systematically learn about high-quality information obtained from the internet so that they can accurately use and deliver e-health information. This is because the acquisition of accurate e-health literacy can improve health behavior in nursing students.

In sum, it can be said that self-care agency, e-health literacy, social media use for health information, and health information-seeking behaviors should be improved in order to promote nursing students’ health-promoting behaviors. Thus, it is necessary to develop and apply systematic educational strategies and educational programs. Future studies should investigate nursing students’ perspectives on online health information-seeking behaviors and develop academic strategies and educational programs to improve health-promoting behaviors.

### 4.3. Limitations and Recommendations

This study has some limitations. First, for convenience, this study was conducted on nursing students from three universities. Therefore, our findings should not be generalized to all nursing students. Second, since the cross-sectional design made no inferences about causality, experimental or longitudinal studies are needed to evaluate causal directionality in future studies. Finally, although other types of personal resources such as physiological and psychological variables mediate health-promoting behaviors, the current study only tested three mediators. Therefore, future studies should consider other diverse mediators in order to understand the relationship between e-health literacy and health-promoting behaviors, and to precisely determine how health-promoting behaviors are influenced. 

Nevertheless, this study’s findings represent the first attempt to examine the relationship between e-health literacy and health-promoting behaviors in nursing students. Additionally, the study is of significance because self-reported e-health literacy, online health information-seeking behaviors, self-care agency, and health-promoting behaviors in nursing students were thoroughly examined. The acquired data are useful in exploring effective ways to enhance health-promoting behavior.

### 4.4. Contributions and Practical Implications for Nursing Practice

Our study may prove helpful for future research studying the causal relationship between e-health literacy and health-promoting behaviors, considering social media use for health information, online health information-seeking behaviors, and self-care agency in nursing students. The results of this study have important practical implications because e-health literacy could help enhance health-promoting behaviors directly and indirectly via the mediating path of social media use for health information, online health information-seeking behaviors, and self-care agency. The findings of this study have shown that high levels of e-health literacy are more likely to promote searching for health information on the internet, which affects self-care agency, leading to better health-promotion behavior. As a result, nurses could support patients using health information retrieved from the internet and improve the quality of care using e-health technology skills.

## 5. Conclusions

The current study found that the direct effect of e-health literacy on health-promoting behaviors of nursing students was statistically significant. The serial multiple mediation effects of social media use for health information, online health information-seeking behaviors, and self-care agency on predicting health-promoting behaviors from e-health literacy were found to be statistically significant. The positive effect of e-health literacy on health-promoting behaviors appears to be partially mediated by the effect of e-health literacy on better social media use, as users of popular social networking sites had greater online health information-seeking behaviors, which improved self-care agency. This result suggests that public health education programs should be developed to improve e-health literacy in nursing students. Additionally, health interventions to improve health-promoting behaviors should be developed, and research evaluating the effect of such interventions should be conducted.

## Figures and Tables

**Table 1:** Descriptive Statistics (*n* = 558).

Characteristics	Mean (SD)	Range	*n* (%)
Age (year)	20.3 (2.2)	17–43	
Gender			
Male			65 (11.6)
Female			493 (88.4)
Grade			
Freshman			164 (29.4)
Sophomore			129 (23.1)
Junior			130 (23.3)
Senior			135 (24.2)
Religion			
Christianity			142 (25.4)
Roman Catholicism			44 (7.9)
Buddhism			33 (5.9)
None			339 (60.8)
Time spent using the internet per week (days)			
1 or 2 days			47(8.4)
3 or 4 days			94(16.8)
More than 4 days			417(74.7)
Time spent using the internet per day (hours)			
<2			173 (31.0)
2 to <3			142 (28.7)
3 to <4			99 (20.0)
≥4			101 (20.4)
Health status			
Good			309 (55.4)
Neutral			211 (37.8)
Bad			38 (6.8)
Self-care agency	156.17 (20.17)	105–204	
E-health literacy	30.53 (5.20)	15–40	
Social media use for health information	2.22 (1.10)	0–4	
Online health information-seeking behaviors	36.61 (9.93)	13–65	
Health-promoting behaviors	2.50 (0.44)	1.1–4.0	
Health responsibility	2.17 (0.58)	1–4	
Physical activity	2.10 (0.72)	1–4	
Nutrition	2.18 (0.61)	1–4	
Spiritual growth	2.87 (0.61)	1.1–4.0	
Interpersonal support	3.15 (0.57)	1.2–4.0	
Stress management	2.55 (0.59)	1.1–4.0	

**Table 2:** Correlations between self-care agency, e-health literacy, social media use for health information, online health information-seeking behaviors, and HPB (*n =* 558).

	r (*p*)				
Variables	Self-Care Agency	E-Health Literacy	Social Media Use for Health Information	Online Health Information- Seeking Behaviors	HPB
Self-care agency	—				
E-health literacy	0.45 (<0.001)	—			
Social media use for health information	0.26 (<0.001)	0.26 (<0.001)	—		
Online health information-seeking behaviors	0.29 (<0.001)	0.25 (<0.001)	0.36 (<0.001)	—	
HPB	0.64 (<0.001)	0.37 (<0.001)	0.25 (<0.001)	0.39 (<0.001)	—

Note. HPB = Health-Promoting Behaviors.

**Table 3:** Total, direct, and indirect effects in the multiple mediator model.

Model	Effect	SE	t	*p*	95% BC CI
Total effect of e-HL on HPB	1.61	0.16	9.61	<0.001	1.28, 1.94
Direct effect of e-HL on HPB	0.30	0.15	1.97	0.048	0.001, 0.60
Total indirect effect	1.31	0.13			1.04, 1.58
Indirect effect via social media use for health information	0.01	0.04			−0.05, 0.10, ns
Indirect effect via social media use for health information, OHISB	0.07	0.02			0.03, 0.11
Indirect effect via social media use for health information and self-care agency	0.06	0.02			0.01, 0.12
Indirect effect via social media use for health information, OHISB, and self-care agency	0.03	0.01			0.01, 0.05
Indirect effect via OHIS	0.14	0.04			0.06, 0.24
Indirect effect via self-care agency	0.91	0.11			0.70, 1.14
Indirect effect via OHIS and self-care agency	0.06	0.02			0.02, 0.11

Note. e-HL = e-health literacy; HPB = Health-Promoting Behaviors; OHISB = Online Health Information-Seeking Behaviors; BC CI = bias-corrected confidence interval; ns = not significant.

## Data Availability

The data presented in this study are available on request from the corresponding author. The data are not publicly available for privacy reasons.
